# Effect of *Fagonia Arabica *(Dhamasa) on in vitro thrombolysis

**DOI:** 10.1186/1472-6882-7-36

**Published:** 2007-11-06

**Authors:** Sweta Prasad, Rajpal Singh Kashyap, Jayant Y Deopujari, Hemant J Purohit, Girdhar M Taori, Hatim F Daginawala

**Affiliations:** 1 Biochemistry Research Laboratory, Central India Institute of Medical Sciences, 88/2, Bajaj Nagar, Nagpur-440010, Maharashtra, India; 2 Environmental Genomics Unit, NEERI, Nehru Marg, Nagpur-440020, India

## Abstract

**Background:**

Atherothrombotic diseases such as myocardial or cerebral infarction are serious consequences of the thrombus formed in blood vessels. Thrombolytic agents are used to dissolve the already formed clots in the blood vessels; however, these drugs have certain limitations which cause serious and sometimes fatal consequences. Herbal preparations have been used since ancient times for the treatment of several diseases. Herbs and their components possessing antithrombotic activity have been reported before; however, herbs that could be used for thrombolysis has not been reported so far. This study's aim was to investigate whether herbal preparations (aqueous extract) possess thrombolytic activity or not.

**Methods:**

An in vitro thrombolytic model was used to check the clot lysis effect of six aqueous herbal extracts viz., *Tinospora cordifolia*, *Rubia cordifolia*, *Hemidesmus indicus, Glycyrrhiza glabra Linn*, *Fagonia Arabica *and *Bacopa monnieri Linn *along with Streptokinase as a positive control and water as a negative control.

**Results:**

Using an in vitro thrombolytic model, *Tinospora cordifolia*, *Rubia cordifolia*, *Hemidesmus indicus, Glycyrrhiza glabra *Linn, *Fagonia Arabica *and *Bacopa monnieri *Linn showed 19.3%, 14.5%, 20.3%, 17.8%, 75.6% and 41.8% clot lysis respectively . Among the herbs studied *Fagonia arabica *showed significant % of clot lysis (75.6%) with reference to Streptokinase (86.2%).

**Conclusion:**

Through our study it was found that Dhamasa possesses thrombolytic properties that could lyse blood clots in vitro; however, in vivo clot dissolving properties and active component(s) of Dhamasa for clot lysis are yet to be discovered. Once found Dhamasa could be incorporated as a thrombolytic agent for the improvement of patients suffering from Atherothrombotic diseases.

## Background

A blood clot (thrombus) developed in the circulatory system due to failure of hemostasis causes vascular blockage and while recovering leads to serious consequences in atherothrombotic diseases such as myocardial or cerebral infarction, at times leading to death [1]. Thrombolytic agents that include tissue plasminogen activator (t-PA), Urokinase (UK), streptokinase (SK) etc. are used all over the world for the treatment of these diseases. In India, though SK and UK are widely used due to lower cost, [2,3] as compared to other thrombolytic drugs, their use is associated with hyper risk of hemorrhage [4] severe anaphylactic reaction and lacks specificity. Moreover, as a result of immunogenicity multiple treatments with SK in a given patient are restricted [5]. Because of the shortcomings of the available thrombolytic drugs, attempts are underway to develop improved recombinant variants of these drugs [6-10].

Herbal products are often perceived as safe because they are "natural" [11]. In India, in recent years, there is increased research on traditional ayurvedic herbal medicines on the basis of their known effectiveness in the treatment of ailments for which they have been traditionally applied.

Considerable efforts have been directed towards the discovery and development of natural products from various plant and animal sources which have antiplatelet [12,13], anticoagulant [14,15], antithrombotic [16], and thrombolytic activity. Epidemiologic studies have provided evidence that foods with experimentally proved antithrombotic effect could reduce risk of thrombosis. Herbs showing thrombolytic activity have been studied and some significant observations have been reported [17].

The aim of present study was to screen aqueous extracts of various herbs viz., *Tinospora cordifolia *(Guduchi), *Rubia cordifolia *(Manjistha), *Hemidesmus indicus *(Anantmool), *Glycyrrhiza glabra *Linn(Yestimadhu), *Fagonia Arabica *(Dhamasa) &* Bacopa monnieri *Linn (Brahmi) for their clot lysis property (thrombolytic activity) by using an in-vitro procedure.

## Methods

### Streptokinase (SK)

To the commercially available lyophilized SK vial (Polamin Werk GmbH, Herdecke, Germany) of 15, 00,000 I.U., 5 ml sterile distilled water was added and mixed properly. This suspension was used as a stock from which 100 μl (30,000 I.U) was used for in vitro thrombolysis.

### Specimen

Whole blood (4 ml) was drawn from healthy human volunteers (*n *= 20) without a history of oral contraceptive or anticoagulant therapy (using a protocol approved by the Institutional Ethics Committee of Central India Institute of Medical Sciences, Nagpur). 500 μl of blood was transferred to each of the eight previously weighed microcentrifuge tubes to form clots.

### Herbal preparation

Commercially available total extract of herbs, *Tinospora cordifolia *(guduchi), *Rubia Cordifolia *(manjistha), *Hemidesmus indicus *(Anantmool), *Glycyrrhiza glabra Linn *(yestimadhu) *Fagonia arabica *(Dhamasa) and *Baccopa monnieri *(Linn) (brahmi) were purchased from Innocon Foods (Pune, India). The multiple solvent (methanol: isopropyl alcohol: acetone) extraction procedure was used to prepare the extract by the supplier. 100 mg extract was suspended in 10 ml distilled water and the suspension was shaken vigorously on a vortex mixer. The suspension was kept overnight and decanted to remove the soluble supernatant, which was filtered through a 0.22-micron syringe filter. 100 μl of this aqueous preparation of herbs was added to the microcentrifuge tubes containing the clots to check thrombolytic activity.

### Clot lysis

Experiments for clot lysis were carried as reported earlier [18]. In brief, 4 ml venous blood drawn from healthy volunteers was distributed in eight different pre weighed sterile microcentrifuge tube (0.5 ml/tube) and incubated at 37°C for 45 minutes. After clot formation, serum was completely removed without disturbing the clot and each tube having clot was again weighed to determine the clot weight (clot weight = weight of clot containing tube – weight of tube alone).

To each microcentrifuge tube containing pre-weighed clot, 100 μl of aqueous extract of six herbs (*Tinospora cordifolia*, *Rubia Cordifolia*, *Hemidesmus indicus*, *Glycyrrhiza glabra Linn, Fagonia arabica *&* Bacopa monnieri *(Linn)) was added separately. As a positive control, 100 μl of SK and as a negative non thrombolytic control, 100 μl of distilled water were separately added to the control tubes numbered. All the tubes were then incubated at 37°C for 90 minutes and observed for clot lysis. After incubation, fluid released was removed and tubes were again weighed to observe the difference in weight after clot disruption. Difference obtained in weight taken before and after clot lysis was expressed as percentage of clot lysis. The experiment was repeated 20 times with the blood samples of 20 volunteers.

### Statistical analysis

The significance between % clot lysis by Streptokinase and herbal extract by means of weight difference was tested by the paired t-test analysis. Data are expressed as mean ± standard deviation.

## Results

**Figure 1:**
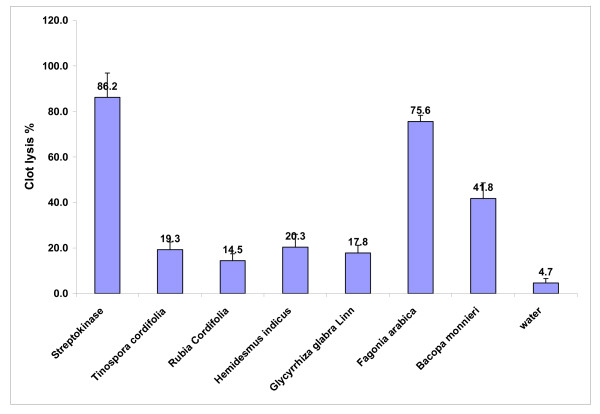
**Clot lysis by Streptokinase, water and various herbal preparations**. Effects of drugs on dissolution of clots prepared from blood of normal individuals. Maximum clot lysis was observed in clot treated with streptokinase (SK). Among herbal drugs *Fagonia arabica *(Dhamasa) showed 75.6% clot lysis. *Bacopa monnieri *Linn (Brahmi) showed 41.8% clot lysis. Water (as a negative control) showed 4.7% clot lysis. With other herbs negligible clot lysis was observed.

Addition of 100 μl SK, a positive control (30,000 I.U.) to the clots along with 90 minutes of incubation at 37°C, showed 86.2% clot lysis. Clots when treated with 100 μl sterile distilled water (negative control) showed only negligible clot lysis (4.7%). The mean difference in clot lysis percentage between positive and negative control was very significant (p value < 0.0009). After treatment of clots with 100 μl of *Tinospora cordifolia, Rubia cordifolia, Hemidesmus indicus, Glycyrrhiza glabra Linn*, negligible clot lysis i.e., 19.3%, 14.5%, 20.3%, 17.8% respectively was obtained but mean of percentage of clot lysis was more than water. But when 100 μl *Fagonia arabica *and *Bacopa monnieri *were added to two different clots, 75.6% and 41.8% clot lysis was obtained respectively and when compared with the negative control (water) the mean clot lysis % difference was significant (p value < 0.0001 & = 0.0023 respectively). Percent clot lysis obtained after treating clots with different herbs and appropriate controls is shown in Figure 1. Statistical representation of the effective clot lysis percentage by six herbal preparations, positive thrombolytic control (Streptokinase) and negative control (sterile distilled water) is tabulated in Table 1.

**Table 1:** Effect of six different herbal extracts (10 mg/ml) on in vitro clot lysis.

Herbs/Drugs	Mean ± S.D. (Clot lysis %)	P value when compared to negative control (water)
Streptokinase	86.2 ± 10.7	< 0.0009
*Fagonia arabica*	75.6 ± 2.7	< 0.0001
*Tinospora cordifolia*	19.3 ± 3.4	0.0045
*Rubia cordifolia*	14.5 ± 3	0.0231
*Hemidesmus indicus*	20.3 ± 5.7	0.0072
*Glycyrrhiza glabra Linn*	17.8 ± 3.4	0.0115
*Bacopa monnieri*	41.8 ± 6.8	0.0023

Statistical representation of the effective clot lysis percentage by six herbal preparations, positive thrombolytic control (Streptokinase) and negative control (sterile distilled water) done by *paired t-test analysis*; clot lysis % is represented as mean ± S.D. and a *p value *< 0.05 was considered as significant.

## Discussion

Herbal preparations are used since ancient times to maintain health and regain healthy state of mind. Advances in phytochemistry and identification of plant compounds, which are effective in curing certain diseases have renewed the interest in herbal medicines. About 30% of the pharmaceuticals are prepared from plants worldwide [19]. A number of studies have been conducted by various researchers to find out the herbs and natural food sources and their supplements having antithrombotic (anticoagulant and antiplatelet) effect and there is evidence that consuming such food leads to prevention of coronary events and stroke [20-23]. There are several thrombolytic drugs obtained from various sources. Some are modified further with the use of recombinant technology [24] in order to make these thrombolytic drugs more site specific and effective. Side effects related to these drugs have been reported that lead to further complications [25]. Sometimes the patients die due to bleeding and embolism [24,26-28].

Herbal preparations, if taken in appropriate dose, can lead to a better option for curing various ailments. Toxicity of plant extract is a major concern of scientists and medical practitioners. Among several methods lethality test has been successfully used to biomonitor the isolation of cytotoxic, antimalarial, insecticidal and antifeedants compounds from plant extracts [29]. In this aspect toxicity of plant extract could be beneficial to mankind however, if taken in higher/lethal dose plant extracts could be harmful [30,31]. Several lethality tests have been designed and one such method is the lethality test wherein Brine Shrimp Lethality (LC50, 24 hr.) test has been used to determine cytotoxicity which has shown toxicity of *Fagonia arabica *to be 900 μg/ml [29].

In our study of thrombolysis we have tried six herbal preparations that are used since ancient times for neuroprotection and for curing vascular diseases (Table 2). For example, *Hemidesmus indicus *was reported to have antithrombotic activity [32]. The roots of *Rubia cordifolia *are used internally in the treatment of abnormal uterine bleeding, as well as for internal and external haemorrhage [33]. *Bacopa monnieri Linn *is used for the treatment of stroke and Alzheimer disease [34]. *Glycyrrhiza glabra Linn *was reported to have antiplatelet and anti-inflammatory activity [35]. *Fagonia arabica *is known to have blood purifying property [36]. *Tinospora cordifolia *along with *Fagonia arabica *and *Rubia cordifolia *was reported to exert neuroprotection [37]. The aim of this study was to check if these herbal preparations posses clot lytic activity.

**Table 2:** List of herbal preparations used in the study and related medicinal uses.

**S. No.**	**Herbal preparation**	**Medicinal use**
1	*Fagonia arabica*	blood purifier, deobstruent
2	*Bacopa monnieri*	treatment of stroke and Alzheimer disease
3	*Tinospora cordifolia*	neuroprotection
4	*Hemidesmus indicus*	antithrombotic activity
5	*Glycyrrhiza glabra Linn*	antiplatelet and anti-inflammatory activity
6	*Rubia cordifolia*	used internally in the treatment of abnormal uterine bleeding, as well as for internal and external haemorrhage

SK, a known thrombolytic drug [38] is used as a positive control. Water, on the other hand, was selected as a negative control. The comparison of positive control with negative control clearly demonstrated that clot dissolution does not occur when water was added to the clot. The percentage of clot lysis by both these controls differ significantly as the p value was 0.0009 (test was extremely significant). Encouraged by the results obtained through the clot lysis activity by SK and with water as a negative control we tried six herbal preparations in the same manner. When compared with the clot lysis percentage obtained through water (negative control), a significant thrombolytic activity was observed after treating the clots with *Fagonia arabica *extract (75.6%; p value < 0.0001).

*Fagonia arabica *belongs to Zygophyllaceae family [39] and is known as 'Kharasan' thorn in English and by a common name of "Dhamasa" in India. It is a green shrub of 1 to 3 feet height, found on calcareous rocks distributed throughout the Mediterranean region of South Africa, Afghanistan, India (Rajasthan, northwest Punjab, and Western India), and Pakistan (Sindh, Punjab, North-West Frontier Province, NWFP) [40]. The whole plant is used for medical purpose; popularly known in hilly areas as a fever remedy source. Infusion is effective as cooling agent in stomatitis. It is known to purify blood and also acts as a deobstruent [36]. It is also used for skin diseases, small pox and for endothermic reaction in the body [41]. The twigs of the plant are used as remedy for snake bite and also applied externally as paste on tumors and for the swellings of neck [39-41].

Interestingly *Bacopa monnieri Linn *exhibited nearly 50% of clot lysis. So far, researchers have reported the use of nitric oxide from *Bacopa monnieri Linn *in the treatment of stroke and Alzheimer's sufferers. Moreover, it has been valued as a cardiac, nerve and brain tonic [34].

There are few plant extracts/products which have been identified to have fibrinolytic activity. These are *Lumbricus rubellus *[42], *Pleurotus ostreatus *[43], *Spirodela polyrhiza *[44], *Flammulina velutipes *[45], and *Ganoderma lucidum *[46], Ginger (*Zingiber officinale*) [47], Garlic (*Allium sativum*) [48], Saffron {Crocus sativus Linn (indraceae)} [49] as well as from Bacillus sp. in Korean and Japanese fermented foods, chungkook-jang [50] and natto [51,52] respectively. Most of these are enzymes which closely resembles serine proteases like plasmin as these are relatively specific to fibrin or fibrinogen as a protein substrate. In general, blood clots are formed from fibrinogen by thrombin and are lysed by plasmin, which is activated from plasminogen by a tissue plasminogen activator. However, there is one marine brown algae product called Seanol (phlorotannin – active compound), have a unique property in promotion of dissolution of intravascular blood clot via antiplasmin inhibition [53].

*Fagonia arabica *is known to have antibacterial activity [29] which is done against two strains of bacteria notably, *Escherichia coli *and *Staphylococcus aureus *[54]. However, there are reports of bacterial contaminants of plants which have plasminogen receptors that bind plasminogen. Cell surface bound plasminogen is easily activated to plasmin, which could lead to fibrinolysis [55]. Bacterial plasminogen activator: staphylokinase, streptokinase, act as cofactor molecules that contribute to exosite formation and enhance the substrate presentation to the enzyme. Staphylokinase activates plasminogen to dissolve clots, also destroys the ECM and fibrin fibers that hold cells together [56,57].

In context of the above discussion it would be interesting to investigate the causative components/mechanism for clot lysis by *Fagonia arabica *and *Bacopa monnieri Linn *extract with respect to its toxicity, bacterial contamination, plasmin activation, inhibitors of antiplasmins, or phytochemicals/secondary metabolites.

## Conclusion

In conclusion, on the basis of beneficial effect of *Fagonia arabica *(Dhamasa) in the literature and our own results of the experiments in the extract of same herb, *Fagonia arabica *lyses blood clots in vitro; however, in vivo clot dissolving property and active component(s) of Dhamasa for clot lysis are yet to be found out. Once found Dhamasa may be incorporated as a thrombolytic agent for the improvement of the patients suffering from Atherothrombotic diseases.

## Competing interests

The author(s) declare that they have no competing interests.

## Authors' contributions

SP carried out the study design, experiments, data collection, data interpretation, literature search and manuscript preparation. RSK and HJP participated in study design, experiments, data interpretation, and literature search and manuscript preparation. JYD provided all the details on herbs and ayurvedic literature. GMT provided assistance in preparation of the manuscript, data interpretation, study design and collecting funds. HFD supervised the study design, statistical analysis, data interpretation, manuscript preparation and literature search. All authors read and approved the final version of the manuscript.

## Pre-publication history

The pre-publication history for this paper can be accessed here:


